# Physicians' Perceptions on the usefulness of contextual information for prioritizing and presenting alerts in computerized physician order entry systems

**DOI:** 10.1186/1472-6947-12-111

**Published:** 2012-10-02

**Authors:** Martin Jung, Daniel Riedmann, Werner O Hackl, Alexander Hoerbst, Monique W Jaspers, Laurie Ferret, Kitta Lawton, Elske Ammenwerth

**Affiliations:** 1 Institute of Health Informatics, UMIT - University for Health Sciences, Medical Informatics and Technology, Hall in Tirol, Austria; 2 Department of Medical Informatics, Academic Medical Center - University of Amsterdam, Amsterdam, The Netherlands; 3 Pharmacy Department, Hospital of Denain, Denain, France; 4 EA2694, University Hospital of Lille, Lille, France; 5 Corporate IT Capital Region, Copenhagen, Denmark

**Keywords:** CPOE, Computerized physician order entry, CDS, Computerized decision support, Contextualization, Clinical context, Alert fatigue, Alert overload, Physician survey

## Abstract

**Background:**

One possible approach towards avoiding alert overload and alert fatigue in Computerized Physician Order Entry (CPOE) systems is to tailor their drug safety alerts to the context of the clinical situation. Our objective was to identify the perceptions of physicians on the usefulness of clinical context information for prioritizing and presenting drug safety alerts.

**Methods:**

We performed a questionnaire survey, inquiring CPOE-using physicians from four hospitals in four European countries to estimate the usefulness of 20 possible context factors.

**Results:**

The 223 participants identified the *‘*s*everity of the effect’* and the *‘clinical status of the patient’* as the most useful context factors. Further important factors are the *‘complexity of the case’* and the *‘risk factors of the patient’*.

**Conclusions:**

Our findings confirm the results of a prior, comparable survey inquiring CPOE researchers. Further research should focus on implementing these context factors in CPOE systems and on subsequently evaluating their impact.

## Background

### Medication errors and adverse drug events

According to estimates from the World Health Organization and the European Commission, approximately 10% of all patients in developed countries are harmed by errors or adverse events during their hospitalization care [1,2]. Medication-related events are among the most common adverse events [3], of which, according to the Institute of Medicine, at least 25% are preventable [4]. Moreover, the costs associated with additional hospitalization are high and the economic benefits of improving patient safety are likewise compelling [1]. Therefore, actions to ensure medication safety have become major global public health issues [1,5].

The Council of Europe defines a medication error as *“any preventable event that may cause or lead to inappropriate medication use or patient harm while the medication is in the control of the health care professional, patient or consumer”*[6] and an adverse drug event (ADE) as *“any injury occurring during the patient’s drug therapy and resulting either from appropriate care, or from unsuitable or suboptimal care”*[6]. ADEs related to a medication error are considered preventable [6].

### CPOE systems and alert fatigue

Of the preventable ADEs, 56%-71% occur during drug prescription [7,8]. Computerized Physician Order Entry (CPOE) systems have the potential to reduce medication error rates as well as ADEs [4,9-12]. CPOE systems can be combined with computerized decision support (CDS). Kuperman et al. distinguish between basic (e.g. offers drug-drug interaction checks) and advanced decision support (e.g. offers drug-disease contraindication checking) [13]. CPOE systems with advanced CDS have proven to be more effective in decreasing medication errors compared to CPOE systems with basic or without CDS [9].

However, CPOE systems with CDS tend to produce a high number of drug safety alerts and often suffer from poor signal-to-noise ratios [14-18]. This burden of unspecific alerts for the physicians is called alert overload and can consequently lead to alert fatigue. Alert fatigue is described by van der Sijs as *“the mental state that is the result of alerts consuming too much time and mental energy, which can cause relevant alerts to be unjustifiably overridden along with clinically irrelevant ones”*[19]. Furthermore, a systematic review from 2006 showed that in 49%-96% of the cases, CPOE/CDS alerts are overridden by the clinicians, which can indirectly impair patient safety [20].

### Contextualization of CPOE alerts

One possible approach to reducing alert fatigue is to filter irrelevant and non-urgent alerts and, furthermore, tailor the alerts to a specific patient [20]. A similar approach was pursued by the European PSIP (Patient Safety through Intelligent Procedures in Medication) project, which aimed to contextualize the delivery of alert information based on antecedent semi-automatic ADE detection using data and semantic mining techniques [21].

**Figure 1:**
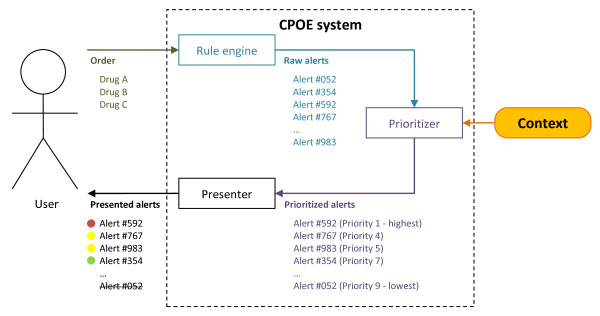
**A contextaware CPOE system.** Depending on the prescription, the rule engine of the CDS system generates raw alerts (e.g. drug-drug interaction between acetylsalicylic acid and an anticoagulant). These raw alerts are then prioritized based on context information (e.g. the dose, the age of the patient, any co-medication, or information on the user or clinical department). Afterwards, the alerts are presented differently to the user according to their priority (e.g. life-threatening alerts interrupt the prescribing process and cannot be overridden). Alert IDs are chosen arbitrarily. Figure by Riedmann and Jung published originally in [23].

In the course of the PSIP project, Riedmann, Jung et al. propounded a concept of a CPOE system that prioritizes and presents drug safety alerts depending on the clinical context. The underlying concept is based on the definition of context provided by Dey: *“Context is any information that can be used to characterize the situation of an entity. An entity is a person, place, or object that is considered relevant to the interaction between a user and an application, including the user and applications themselves”*[22]. Consequently, a context-aware system *“uses context to provide relevant information and/or services to the user, where relevancy depends on the user’s task”*[22]. Figure 1 schematically illustrates the concept of a context-aware CPOE system that uses information about the context of the clinical situation in order to prioritize alerts and to offer a tiered alert presentation to the user (figure published originally in [23]).

**Figure 2:**
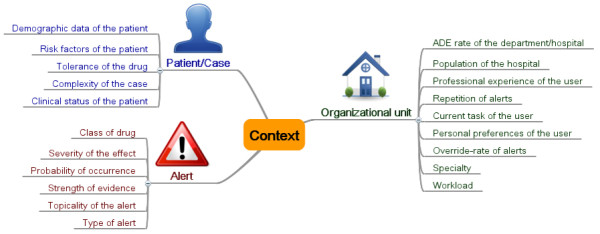
Mind map of the context factors grouped into three categories.

According to this concept, Riedmann, Jung et al. compiled a set of 20 potential context factors on the basis of a literature search and of qualitative expert interviews. The context factors were then hierarchically structured into three main axes: 1.) Characteristics of the organizational unit; 2.) Characteristics of the patient or case; 3.) Characteristics of the alert (see Figure 2, published originally in [23]). For definitions and examples of each context factor, see Additional file 1.

### Problem

To determine which of these context factors were useful to support this concept of context-aware CPOE systems, Riedmann et al. conducted a Delphi study with international CPOE researchers [24]. The obtained results reflect the opinions of publishing CPOE researchers; more than 50% of them held a university perspective and only a quarter of the participants were practicing physicians [24].

The objective of the present study was to investigate the viewpoints of the clinical CPOE users in a survey, as the results of the Delphi study may not reflect their opinions.

At the moment, most physicians have to deal with often annoying CPOE alerts and mostly do not have experience with contextualized alert presentation. Therefore, it may be difficult for them to foresee which approaches may be useful and which not. Answers in a survey may thus not reflect actual behavior in the future. However, surveys are an important and efficient way to obtain a first understanding of what physicians consider useful to reduce alert overload.

### Study question

What are the perceptions of physicians on the usefulness of clinical context information for prioritizing and presenting drug safety alerts?

## Methods

### Study context

The study was carried out with a convenience sample of European hospitals in Amsterdam, Copenhagen, Denain and Sofia (see Table 1). All hospitals implemented CPOE/CDS systems for prescribing drugs. In Copenhagen, Denain and Amsterdam, commercial CPOE products were currently in use, while the hospital in Sofia developed its own CPOE system together with a commercial vendor. All CPOE systems, except for the CPOE system in Denain, provide automatic alerts to the physicians; this means that alerts are automatically triggered and presented to the user during drug prescription. Physicians in Denain are only alerted optionally if they explicitly ask the system for advice (e.g. by clicking a “check prescription” button). According to the classification provided by Kuperman [13], all CPOE systems possess basic CDS functionalities. None of the CPOE systems offered advanced CDS functionalities. Table 2 shows the details of the CPOE systems in use.

**Table 1:** Key data of 2010 of the participating study hospitals

**City** (**Country**)	**Participating hospital**(**s**)	**Type of hospital**	**Number of beds**	**Number of employees**
**Amsterdam** (The Netherlands)	AMC Amsterdam	University hospital	1,002	6,957
**Copenhagen** (Denmark)	Glostrup Hospital, Herlev Hospital, Hillerød Hospital	Regional hospitals	1,407	11,800
**Denain** (France)	CH Denain	Regional hospital	600	1,000
**Sofia** (Bulgaria)	USHATE Sofia	Specialized university hospital for Endocrinology	109	170

**Table 2:** Details of the CPOE systems in use

	**Amsterdam**	**Copenhagen**	**Denain**	**Sofia**
**CPOE name**	Medicator/ESV	EPM	CPOE module of DxCare	Medica
**CPOE vendor**	iSoft	Accure/IBM	Medasys	Home-grown/Macrosoft
**Year of CPOE introduction**	2004	Glostrup: 2009	2003	2010
		Herlev: 2007		
		Hillerød: 2006		
**Type of alerting**	Automatic alerts^1^	Automatic alerts^1^	Optional alerts^2^	Automatic alerts^1^
**Basic CDS functionalities according to Kuperman**[13]				
**Drug**-**allergy checking**		X		
**Basic dosing guidance**	X		X	X
**Formulary decision support**				
**Duplicate therapy checking**	X	X		
**Drug**-**drug interaction checking**	X	X	X	
**Advanced CDS functionalities according to Kuperman**[13]				
None implemented (advanced dosing guidance, guidance for medication-related laboratory testing, drug-disease contraindication checking, drug-pregnancy checking).				

^1^Automatic alerts are triggered by the system and pop up automatically. ^2^Optional alerts are triggered by the user and only pop up if the user explicitly asks for advice (e.g. by clicking a “check prescription” button).

### Study design

We performed a one-group cross-sectional quantitative questionnaire survey. We chose an exploratory and observational design to generate new insights into the issue of CPOE alert contextualization from a physician’s point of view.

The study design was presented to the ethics committee at UMIT. A formal approval of the design was not considered necessary by the committee.

### Participants

To meet the requirements of the local study managers, the questionnaires were either available paper-based or online. Copenhagen, Denain and Sofia wanted to use the paper-based version, whereas Amsterdam opted for the electronic questionnaire. In all hospitals except for Copenhagen, full-sample surveys were conducted. In Copenhagen, due to organizational reasons, we drew a convenience sample of participants. As all three participating hospitals from Copenhagen had been using the same CPOE system for approximately the same time period, the data was aggregated to one single dataset. Table 3 provides further information about the sampling.

**Table 3:** Sampling information of the studies in the individual hospitals

**Hospital**	**Type of questionnaire**	**Sampling strategy**	**Addressed departments**	**Number of addressed physicians**
**Amsterdam**	Electronic	Full sample	All departments	998
**Copenhagen**	Paper-based	Convenience sample	Anesthesia (A)	207 (A = 20, IM = 102, GS = 85)
			Internal medicine (IM)	
			Gastro-surgery (GS)	
**Denain**	Paper-based	Full sample	All departments	60
**Sofia**	Paper-based	Full sample	All departments	53

At all the hospitals, the physicians were informed beforehand about the objectives of the survey personally or via e-mail. Local coordinators then took charge of the organizational issues (i.e. recruiting and reminding the physicians as well as distributing and collecting the questionnaires). No medical students were addressed. At all the hospitals except for Amsterdam, the physicians were recruited and reminded personally; in Amsterdam this was done electronically via e-mail.

### Study flow

The questionnaire was constructed and then pre-tested with seven hospital doctors from different specialties. It was then translated completely into Bulgarian and French and partly into Danish and Dutch. The questionnaire was then again pre-tested at each hospital with two or three doctors. The survey was conducted between 2010 and 2011.

### Methods for data acquisition and measurement

The paper-based and the electronic questionnaire (using *LimeSurvey*http://www.limesurvey.org) comprised the following parts:

Part 1: Selecting and ranking of useful context factors

A double-staged rating process was developed in cooperation with a psychologist responsible for survey management at the University Hospital of Innsbruck:

1. All 20 potential context factors were presented to the physicians. They were asked to mark with a cross those factors which they considered useful to filter irrelevant alerts. The order of the statements was randomized to avoid an unintentional ‘serial position effect’, meaning that the first factors in the list are judged differently than the factors listed further down [25].

2. The physicians were then asked to have another look at those factors marked with a cross, to select the five most useful factors and to rank them according to their usefulness.

Part 2: Personal details

We also asked the physicians to amend as free-text further factors that they would consider useful for filtering alerts.

We asked the physicians to provide demographic data about their age, sex, professional role, years of work experience and years of experience with CPOE systems.

Both parts of the questionnaire are shown in the additional file 1.

### Methods for data analysis

We calculated the frequencies for the number of times each factor was marked as ‘useful’ with a cross and generated a heat map.

Furthermore, we calculated a ranking of the most important context factors. We only took those questionnaires into account in which the physicians had correctly followed the ranking instructions. A factor received between 1 and 5 points based on the rank assigned by each physician (i.e. rank 1 (first most useful) = 5 points; rank 5 (fifth most useful) = 1 point). Points were summed up for every factor per hospital and normalized according to the number of physicians per hospital to account for differences in the sample sizes [26]. To determine the most useful factors, we performed a hierarchical clustering over all hospitals using *SPSS v16* (method: average linkage between groups UPGMA - Unweighted Pair Group Method with Arithmetic Mean; distance measure: squared Euclidian distance). To present the outcome, we used a dendrogram.

The answers to the free-text question that were meant to determine further context factors were analyzed by means of quantitative content analysis with inductive category development according to Mayring [27] using the software tool *MaxQDA 2007*. Two researchers independently classified the comments as to whether they were already covered by our set of 20 context factors. All comments that suggested new ideas for further factors were discussed in the research team.

## Results

### Demographic data

A total of 1,380 questionnaires were distributed. 223 completed questionnaires were returned. The return rate was between 7.5% in Amsterdam and 58.5% in Sofia (Table 4). Across all hospitals, the average physician was between 30–39 years old and had worked around 14 years as a doctor. He/she currently fulfilled a professional role at the medium level and had around 4 years of experience with CPOE systems (Table 5).

**Table 4:** Number of distributed questionnaires and valid return rates of the participating hospitals

**Hospital**	**Distributed questionnaires n**	**Valid return n (%)**
**Amsterdam**	998	75 (7.5%)
**Copenhagen**	207	91 (44%)
**Denain**	60	26 (43.3%)
**Sofia**	53	31 (58.5%)

**Table 5:** Demographic data of the respondents

	**Amsterdam n (%)**	**Copenhagen n (%)**	**Denain n (%)**	**Sofia n (%)**	**Total n (%)**
**Sex**					
Male	38 (50.7%)	52 (57.1%)	16 (61.5%)	10 (32.3%)	116 (52.0%)
Female	30 (40%)	34 (37.4%)	10 (38.5%)	21 (67.7%)	95 (42.6%)
No statement/Missing answer	7 (9.3%)	5 (5.5%)	0 (0%)	0 (0%)	12 (5.4%)
**Age**					
< 29 years	1 (1.3%)	12 (13.2%)	8 (30.8%)	4 (12.9%)	25 (11.2%)
30-39 years	**37** (**49**.**3**%)	**30** (**33**%)	4 (15.4%)	**14** (**45**.**2**%)	**85** (**38**.**1**%)
40-49 years	16 (21.3%)	18 (19.8%)	**8** (**30**.**8**%)	5 (16.1%)	47 (21.1%)
50-59 years	9 (12%)	20 (22%)	3 (11.5%)	2 (6.5%)	34 (15.2%)
> 59 years	7 (9.3%)	3 (3.3%)	3 (11.5%)	6 (19.4%)	19 (8.5%)
No statement/Missing answer	5 (6.7%)	8 (8.8%)	0 (0%)	0 (0%)	13 (5.8%)
**Professional role**					
Low level ^1^	25 (33.3%)	9 (9.9%)	6 (23.1%)	12 (38.7%)	52 (23.3%)
Medium level ^2^	**41** (**54**.**7**%)	**38** (**41**.**8**%)	2 (7.7%)	**5** (**16**.**1**%)	**86** (**38**.**6**%)
High level ^3^	3 (4.0%)	36 (39.6%)	**14** (**53**.**9**%)	12 (38.7%)	65 (29.1%)
Other role	1 (1.3%)	2 (2.2%)	3 (11.5%)	0 (0%)	6 (2.7%)
No statement/Missing answer	5 (6.7%)	6 (6.6%)	1 (3.8%)	2 (6.4%)	14 (6.3%)
**Years working as a doctor**					
Mean (± STD)	14.1 (± 10.2)	13.1 (± 10.8)	16 (± 12.4)	16.4 (± 13.4)	14.3 (± 11.2)
No statement/Missing answer	7 (9.3%)	9 (10%)	4 (15.4%)	0 (0%)	20 (9.0%)
**Years working with a CPOE system**					
Mean (± STD)	5.1 (± 2.8)	3 (± 1.6)	4.8 (± 2.8)	3.1 (± 3.9)	4 (± 2.7)
No statement/Missing answer	8 (10.7%)	11 (12.1%)	0 (0%)	10 (32.2%)	29 (13.0%)

^1^ AGNIOS/AIOS (Amsterdam), Basislæge (Copenhagen), Interne (Denain), Стажант/Специализант/Докторант (Sofia).

^2^ Specialist (Amsterdam), Reservelæge (Copenhagen), Assistant (Denain), Лекар ординатор (Sofia).

^3^ Afdelingshoofd (Amsterdam), Overlæge (Copenhagen), Médecin titulaire (Denain), научнопреподавателски кадри (Sofia).

Absolute (n) and relative (%) values are presented; medians are highlighted in bold.

### Study findings

**Figure 3:**
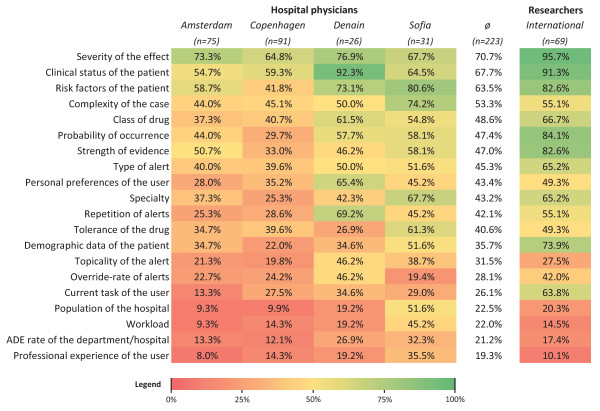
**Heat map of the context factors.** The percentage of physicians in the study hospitals who found a context factor useful to prioritize and filter alerts is shown. An additional column presents the opinion of the CPOE researchers on the same question, obtained by a Delphi study [24]. Colors gradually vary from green (100%) to red (0%). The heat map is sorted descending according to the average frequency per context factor by the physicians.

The heat map in Figure 3 presents the perceived usefulness of the context factors, i.e. the number of physicians who found a context factor would be useful for prioritizing and filtering irrelevant alerts. We added a column for the results from the previous Delphi study, where 69 CPOE researchers were asked about the same matter [24].

**Figure 4:**
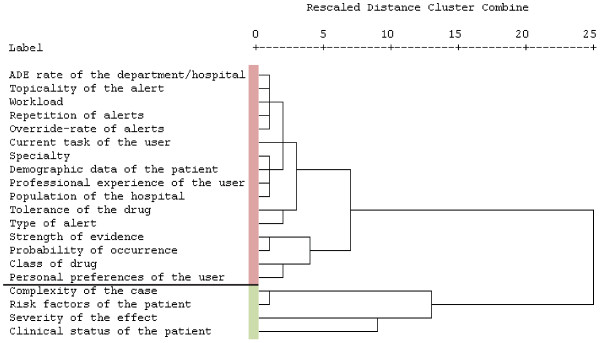
**Dendrogram of the clustered context factors.** This tree diagram is read from left to right; every factor is one cluster at the beginning. Similar clusters are linked step-wise (vertical lines). Distances are transformed into the range from 0 to 25 preserving the original ratios. The longer the horizontal lines and leaps become, the bigger the dissimilarity becomes, which is where to stop the agglomeration.

The dendrogram in Figure 4 shows the results of the cluster analysis to determine the most useful context factors for prioritizing and presenting drug alert information as perceived by the physicians. The red and green bars indicate the two clusters that are the most distinct from each other. The green cluster contains the elements with the highest ranking values.

From the 22 free-text comments provided by 17 physicians, no new ideas could be derived for additional factors to be taken into account for the contextualization of CPOE alerts. Three suggestions were already covered by our set of context factors, whereas 19 comments addressed other issues, such as complaints about the current CPOE system at the hospital.

## Discussion

### Answer to the study question

Four factors were perceived as useful for filtering irrelevant alerts by a majority of all the participating physicians. According to the clustering of the ranking values, the most useful context factors for prioritizing alerts are *‘*s*everity of the effect’* and *‘clinical status of the patient*’. Further important factors are *‘complexity of the case’* and *‘risk factors of the patient’* (see Figure 4).

### Strengths and weaknesses of the study

The survey was carried out at four European hospitals. In general, the response rates were quite high (43%-59%). The return rate in Amsterdam was significantly lower, which may be due to the fact that the physicians were addressed electronically and not personally as it was done at the other hospitals. In Copenhagen, a potential recruitment bias because of the convenience sampling is possible. Due to organizational reasons, no efforts could be taken in order to ensure homogeneous groups between the hospitals. Due to the sampling strategy and the voluntary nature of this survey, the participants cannot be seen as fully representative for all hospital physicians. It seems that especially more experienced physicians participated (> 14 years of experience; professional role at the medium level). These more experienced physicians should provide sufficient practical knowledge to judge a CPOE system. We see this as a strength of this survey.

The translation of the questionnaire was carried out by non-professional translators who were familiar with the field. A multi-stage process including back-translation was not conducted, but might have ameliorated the quality of translation.

The physicians were presented a list of 20 factors. To avoid ‘serial position effects’, the list of context factors was randomized in two different ways in the paper questionnaire and randomized differently for every physician in the electronic questionnaires.

In Denain and Sofia, the physicians generally considered more factors useful (around 10 useful factors/physician on average) than the ones in Amsterdam and Copenhagen (around 6.5 useful factors/physician on average). This can be seen in the heat map in Figure 3, where the columns of Denain and Sofia appear to be ‘greener’. Furthermore, around 40% of the Danish physicians who returned a completed questionnaire did not assign ranks to the context factors in the second step. This was especially the case at Herlev Hospital, which accounted for around 90% of these cases. In general, hospitals at which the physicians were personally recruited and motivated had higher return and completion rates.

Another potential source of bias is the way in which the factors were explained in the questionnaires. Each factor was described with a short definition and a prime example. The assessment of the factors might have been influenced by the given examples (‘example bias’). This effect was anticipated but could not be obviated as the provision of examples is required to achieve a common understanding of a factor.

### Results in relation to other studies

The underlying context factors are based on a literature review and expert interviews. Therefore, each factor has already been discussed in the literature (for details, see [23]). In addition, the Delphi study by Riedmann et al. [24] provided answers to the question as to which factors might be most beneficial in contextualizing alerts from the CPOE researchers’ point of view.

The hospital physicians and the CPOE researchers agreed on the same two top context factors *‘severity of the effect’* and *‘clinical status of the patient’*. Both factors likewise are strongly supported by the literature, but controversy exists. The factor *‘clinical status of the patient’* is considered important by many authors, such as Sittig et al. [28] and Bates et al. [29], who think that it is important to take more clinical parameters about the development of a patient’s condition (e.g. renal function) into account. However, there is some controversy about the importance of the *‘severity of the effect’* as a context factor: Some authors, such as Kuperman et al. [13], Paterno et al. [30] or Weingart et al. [31], endorse the relevancy of this factor, whereas others discussed this factor more controversially. Van der Sijs et al. [20] warn that presenting alerts with different levels of severity increases the override rates compared to alerts without a tiered presentation. Furthermore, the article concluded that the seriousness of an alert, as perceived subjectively by the physicians, might deviate from established objective seriousness indices such as the Dutch seriousness index [32]. Magnus et al. [16] warned that physicians might only stick to high severity alerts and override all low severity alerts. It seems that the calculation algorithm of the severity index is crucial.

The factor *‘risk factors of the patient’* was rated as the fourth most useful by the researchers and also among the top factors in this study, but it is only seldom mentioned in the literature.

The physicians perceived the factor *‘complexity of the case’* as more beneficial than the researchers (researcher-rank 12). This factor has good, positive support in the literature as well - for example, Sittig et al. stated that clinicians *“were more willing to accept clinical decision support when the patient … had multiple medications or chronic conditions”*[28].

Other factors that were top-rated by the researchers (#3 *‘probability of occurrence’* and #5: *‘strength of evidence’*) were not perceived as beneficial by the physicians to the same extent. The reason could be the more objective, scientific view of the researchers, while the physicians are more concerned with the individual patient and his or her situation.

The context factor *‘professional experience of the user’* was rated lowest in the researcher survey. The factor is mentioned quite frequently in the literature and is often used as a prime example to describe the concept of contextualizing drug safety alerts (e.g. senior physicians receive fewer alerts than junior physicians). Riedmann et al. [24] assumed that this outcome might have been related with the theoretical research background of the researchers and that practicing clinicians might hold different views on the relevance of this factor. However, the physicians in this study also found this factor to be of very low importance.

Other factors that were rated low by the physicians were the *‘workload’* and the *‘ADE rate of the department/hospital’*. Both factors are innovative ways for addressing the issue of alert filtering. However, for that reason, physicians might not have been directly impressed by their potential impact in reducing alert overload yet. Furthermore, exact ADE rates are not easy to track. Manual strategies are either not exhaustive, too time-consuming or not reproducible, and automatic detection is still an area of research [21,33-35].

### Meaning and generalizability of the study

To our knowledge, this is the first study that systematically addresses the opinions of European hospital physicians regarding the contextualization of alerts in CPOE systems. The opinions on the usefulness of the context factors for the prioritization of CPOE alerts seem to be quite comparable between the participating hospitals (cf. Figure 3), even when the physicians are working in different organizations and technical settings.

Several CPOE systems already classify their alerts using ‘traffic lights’ (e.g. highlighting potentially dangerous alerts in red color), but mostly, this classification is solely based on the expected severity of the effect and not on any other context information.

The results of our study show that clinical physicians, as well as CPOE researchers [24], also think that CPOE systems should make use of a broader concept of context and take into account more data in order to optimize alert logistics. Additional information about the patients’ clinical status (e.g. development of certain lab values over time) or other risk factors (e.g. allergies) are seen as useful to contextualize alerts. Furthermore, the complexity of the case (e.g. the number of prescribed drugs) could be used to improve drug interaction alerts, which are often unspecific and, thus, of particular annoyance to physicians [13,28].

### Unanswered and New questions

Our findings can be seen as a first, user-centered viewpoint on the issue of ‘contextualizing CPOE systems’. These results only reflect the opinions of clinical physicians and have not yet been empirically verified by implementing contextualized alerting within CPOE systems.

Furthermore, our results do not reveal how the context factors can be implemented into CPOE systems. Within the mentioned PSIP project, some ideas have already been developed and partly implemented [21].

Except for the factor *‘severity of the effect’*, all top context factors identified in our survey refer to patient-related data. Implementing these factors in CPOE/CDS systems would at first require:

● Access to relevant data on the patient and case in a highly structured form (including diagnosis, drugs, lab values, allergies, medical procedures and orders). This data could come from different clinical information systems and possibly even from other institutions.

● Access to a knowledge base that not only contains drugs, their interactions and contraindications, but also information on the interdependencies with diagnoses, allergies, lab values, planned procedures, etc.

● A presentation module of the CPOE that is able to display alerts differently depending on their prioritization value.

Secondly, if these requirements are met, it must be defined as to when and how the available context information is taken into account. Marcilly et al. [36] proposed an approach to classify situations related to drug prescription and tried to identify the “right moment” of alerting (for example, do not alert if a drug-related lab value is normal and sufficiently monitored).

Further work is essential to putting our still theoretical approach into practice. In reality, more than one context factor might trigger in a specific clinical situation. Hence, it might be necessary to combine certain context factors as proposed in [23]. It is unclear which factors could be combined and which combinations prove to be most beneficial for reducing alert overload and ADE rates. Furthermore, if taking more than one factor into account, one will have to deal with multiple, possibly contradictory prioritization values. It then must be specified as to how to compile a single prioritization value. One must also bear in mind that our factors do not represent orthogonal factors, i.e. they interdepend (e.g. the severity of the effect is highly determined by the clinical status of the patient). These are issues still to be addressed by research.

## Conclusions

In this study, 223 physicians from hospitals in four European countries judged the following factors as most useful for prioritizing alerts: *‘severity of the effect’* and *‘clinical status of the patient*’. Further important factors are *‘complexity of the case’* and *‘risk factors of the patient’*. These findings complement the results of a similar CPOE researcher survey [24]. Some identified differences may be explained by the theoretical, more research-oriented viewpoint of the researchers and the practical, more clinical-oriented viewpoint of the physicians.

Our findings might contribute to the ongoing research tackling medication safety. Our survey provided further insights into how to develop more effective alerting strategies. Further research should also involve CPOE developers and vendors in order to learn how to successfully implement these context factors into the next generation of context-aware CPOE and CDS systems. The impact of these systems should then be evaluated in experimental studies and in clinical practice.

## Abbreviations

ADE: Adverse Drug Event; AMC: Academic Medical Center; CDS: Computerized Decision Support; CPOE: Computerized Physician Order Entry; PSIP: Patient Safety through Intelligent Procedures in Medication; UMIT: University for Health Sciences, Medical Informatics and Technology; UPGMA: Unweighted Pair Group Method with Arithmetic Mean; USHATE: University Specialized Hospital for Active Treatment of Endocrinology.

## Competing interests

The authors declare that they have no competing interests.

## Authors’ contributions

DR, MJ, WOH and EA developed the underlying context model. MJ, DR, WOH, AH and EA conceptualized and designed the study. MJ organized the survey and designed the questionnaires. LF, KL and MWJ translated the questionnaires and organized the conduction of the surveys in their respective hospitals. MJ performed the analysis and drafted the manuscript. All authors contributed to the manuscript, critically revised the drafts and approved the final manuscript.

## Pre-publication history

The pre-publication history for this paper can be accessed here:

http://www.biomedcentral.com/1472-6947/12/111/prepub

## Supplementary Material

Additional file 1 **Sample questionnaire.** Excerpt of the English version of the questionnaire used in Denain. *DxCare* is the name of the local CPOE system. Click here for file
