# Supplementary material for: Physicians' Perceptions on the usefulness of contextual information for prioritizing and presenting alerts in computerized physician order entry systems
Source: BMC Med Inform Decis Mak. 2012 Oct 2;12:111. doi: 10.1186/1472-6947-12-111 (PMC3522054; doi:10.1186/1472-6947-12-111)
Supplement: Additional file 1 — Sample questionnaire. Excerpt of the English version of the questionnaire used in Denain. DxCare is the name of the local CPOE system. [file 1472-6947-12-111-S1.pdf]

Part 1: Filtering of medication safety alerts

In some hospitals, systems for prescribing drugs already provide automatic medication safety alerts. They tend to produce a large number of alerts that may not be relevant for a given clinical situation. In your opinion: **Which are useful factors to filter irrelevant alerts?** Please read the following list of potential factors and **mark** those factors **with a cross** that you consider useful.

Factor	Useful (X)
**1. Population of the hospital**<br>The epidemiological characteristics of the population of patients from the geographical catchment area of the hospital.<br>Example: Show more alerts for increased risk of liver destruction when the prevalence for liver diseases is high in the area of the hospital.	☐
**2. Workload**<br>The number of patients to care for, the staffing of the department, the duration of the shift or the time of the day.<br>Example: Certain alerts that might be overlooked should be highlighted when the doctor is working for more than 8h.	☐
**3. Complexity of the case**<br>The number of clinical conditions and multi-morbidities of a patient or the number of applied drugs.<br>Example: Certain alerts that might be overlooked should be highlighted in case that the patient has more than 5 chronic clinical conditions or if he/she takes more than 5 different drugs at the same time.	☐
**4. ADE rate of the department/hospital**<br>The total number of ADEs, which occur in the department/hospital.<br>Example: If a department/hospital has a low rate of a specific ADE, don't show the corresponding alert.	☐
**5. Risk factors of the patient**<br>A certain genetic disposition, alcoholism, obesity or under-nutrition.<br>Example: Show specific alerts only for alcoholics.	☐
**6. Demographic data of the patient**<br>The sex, age and ethnicity of the patient.<br>Example: Show certain alerts only for patients older than 60 years.	☐
**7. Probability of occurrence**<br>The probability of occurrence of the expected ADE.<br>Example: When prescribing an anticoagulant, show an alert only if the probability of internal bleeding is higher than 5%.	☐
**8. Topicality of the alert**<br>How long a certain alert is in the system or the time since the last update of an alert.<br>Example: Highlight alerts that are new in the system.	☐
**9. Work experience of the doctor**<br>Years of working experience, degree and the position in the hierarchy.<br>Example: A senior physician receives fewer alerts than a resident.	☐
**10. Type of alert**<br>The type of drug interaction (e.g. drug-drug interaction, drug-allergy interaction) that may occur.<br>Example: Don't show drug-allergy alerts if allergies are not sufficiently documented in the patient records of the hospital.	☐

**11. Repetition of alerts**<br>The number of times a specific alert is presented to the doctor.<br>Example: An alert is only shown to a doctor once a day and never twice for the same patient (e.g. renewal of a prescription).	☐
**12. Personal preferences of the doctor**<br>Individual customization of the alerts depending on the doctor's needs or preferences.<br>Example: A doctor can turn off certain alerts if he doesn't want them.	☐
**13. Current task of the doctor**<br>The current step in the medication workflow (prescription, dispensation, administration).<br>Example: Show all alerts during the prescription, but only the most important alerts in the step of administration.	☐
**14. Severity of the effect**<br>The seriousness of the potential effect.<br>Example: Don't show alerts when the expected effect may cause no or only minor patient harm.	☐
**15. Override-rate of alerts**<br>The frequency a specific alert gets overridden by a specific doctor, department or hospital-wide.<br>Example: An alert won't be shown again to a doctor if he/she has already overridden it several times.	☐
**16. Special field**<br>The specialist field of the doctor or special field of the department/hospital.<br>Example: A psychiatrist gets different alerts than a surgeon.	☐
**17. Strength of evidence of the effect**<br>The strength of the scientific evidence of a certain effect.<br>Example: Don't show alerts if only one non-randomized study reports this certain effect.	☐
**18. Tolerance of the drug**<br>The case history of the patient shows that he/she tolerates a drug.<br>Example: Don't show alerts for the possible side effects of aspirin, if the patient hasn't developed any of these in a previous case.	☐
**19. Class of drug**<br>The group of the prescribed drug (e.g. narcotics, anticoagulants) relating to the possible damage it may cause.<br>Example: Highlight specific alerts only for classes of drugs with a high ADE potential (e.g. corticosteroids).	☐
**20. Clinical status of the patient**<br>The type of disease, the severity or stadium of the disease or clinical parameters (e.g. lab values).<br>Example: Show specific alerts only if the patient suffers from renal diseases or when a lab value reaches a critical threshold.	☐

Now have a look again at the factors that you considered useful. **Which are the 5 most useful factors?** Please rank them by writing the **number** of the factors in the lines below (Rank 1: first most useful).

Rank 1: __________ (first most useful)

Rank 2: __________

Rank 3: __________

Rank 4: __________

Rank 5: __________ (fifth most useful)

21. In your opinion: Are there other factors that could be used to filter irrelevant alerts?

Part 2: Personal details

1.	Age:	☐ Younger than 20<br>☐ 20-29<br>☐ 30-39	☐ 40-49<br>☐ 50-59<br>☐ Older than 59	☐ No statement
2.	Sex:	☐ Male	☐ Female	☐ No statement
3.	Professional role:	☐ Intern<br>☐ Resident<br>☐ Chief resident	☐ Attending physician<br>☐ Other	☐ No statement
4.	Number of years working as a doctor:		________________ years	☐ No statement
5.	Number of years working with *Dx Care* or comparables systems:		________________ years	☐ No statement
